# Diaphragmatic hernia repair porcine model to compare the performance of biodegradable membranes against Gore-Tex^®^

**DOI:** 10.1007/s00383-023-05584-x

**Published:** 2023-11-24

**Authors:** Marianna Scuglia, Laura P. Frazão, Alice Miranda, Albino Martins, Joana Barbosa-Sequeira, Diana Coimbra, Adhemar Longatto-Filho, Rui L. Reis, Cristina Nogueira-Silva, Nuno M. Neves, Jorge Correia-Pinto

**Affiliations:** 1 Life and Health Sciences Research Institute, School of Medicine, University of Minho, Braga, Portugal; 2 ICVS/3B’s – PT Government Associate Laboratory, Braga/Guimarães, Portugal; 3 3B’s Research Group, I3B’s – Research Institute on Biomaterials, Biodegradables and Biomimetics of University of Minho, AvePark, Parque de Ciência e Tecnologia, Zona Industrial da Gandra, 4805-017 Barco, Guimarães, Portugal; 4 Department of Pediatric Surgery, Hospital de Braga, Braga, Portugal; 5 Department of Pediatric Surgery, Centro Materno Infantil do Norte, Centro Hospitalar Universitário do Porto, Porto, Portugal; 6 Department of Pathology (LIM-14), University of São Paulo School of Medicine, São Paulo, Brazil; 7 Molecular Oncology Research Center, Barretos Cancer Hospital, Barretos, São Paulo, Brazil; 8 Department of Obstetrics and Gynecology, Hospital de Braga, Braga, Portugal

**Keywords:** Gore-Tex^®^, Electrospun fibrous mesh, Decellularized human chorion membrane, Congenital diaphragmatic hernia repair, Thoracoscopic surgery

## Abstract

**Background:**

Patch repair of congenital diaphragmatic hernia (CDH) using Gore-Tex^®^ is associated with infection, adhesions, hernia recurrence, long-term musculoskeletal sequels and poor tissue regeneration. To overcome these limitations, the performance of two novel biodegradable membranes was tested to repair CDH in a growing pig model.

**Methods:**

Twelve male pigs were randomly assigned to 3 different groups of 4 animals each, determined by the type of patch used during thoracoscopic diaphragmatic hernia repair (Gore-Tex^®^, polycaprolactone electrospun membrane-PCLem, and decellularized human chorion membrane-dHCM). After 7 weeks, all animals were euthanized, followed by necropsy for diaphragmatic evaluation and histological analysis.

**Results:**

Thoracoscopic defect creation and diaphragmatic repair were performed without any technical difficulty in all groups. However, hernia recurrence rate was 0% in Gore-Tex^®^, 50% in PCLem and 100% in dHCM groups. At euthanasia, Gore-Tex^®^ patches appeared virtually unchanged and covered with a fibrotic capsule, while PCLem and dHCM patches were replaced by either floppy connective tissue or vascularized and floppy regenerated membranous tissue, respectively.

**Conclusion:**

Gore-Tex^®^ was associated with a higher survival rate and lower recurrence. Nevertheless, the proposed biodegradable membranes were associated with better tissue integration when compared with Gore-Tex^®^.

**Supplementary Information:**

The online version contains supplementary material available at 10.1007/s00383-023-05584-x.

## Introduction

Multiple surgical strategies for congenital diaphragmatic hernia (CDH) have been reported, mainly depending on the size of the diaphragmatic defect and thus susceptible to the surgeon’s individual discretion. Indeed, to standardize procedures, the Congenital Diaphragmatic Hernia Study Group (CDHSG) has developed a classification based on defect size [1]. CDHSG reports have also demonstrated a close association between defect size and morbidity and mortality [1, 2]. In patients with large defects (B and C defects) [1] or complete diaphragmatic agenesis (D), patch repair (PR) and autologous muscular flap remain the only viable surgical options [3]. According to CDHSG, PR accounts for 52.5% of CDH cases [4] and stands consistently as a predictor of adverse outcomes. In fact, PR is associated with infection, adhesions and hernia recurrence, the latest occurring in up to 50% patients aged below 3 years [5]. Long-term sequels are often reported, such as scoliosis (27%) and chest wall deformities (50%) [6], which result from the inability of the prosthetic materials to mimic diaphragmatic properties and expand accordingly in a setting of rapid child growth [7–9].

Thoracoscopic repair of CDH has been performed for the last two decades. When compared to open surgery, it has been associated with fewer post-operative ventilator days, less use of analgesics [10], shorter hospital stays, fewer intra-abdominal complications, better wound cosmesis, lower incidence of subsequent scoliosis and chest deformities [11]. However, a higher recurrence rate after thoracoscopic repair was reported (0–24% vs. 0–11% in open repair) [12]. This was associated with surgery-related technical factors, such as the surgeon learning curve. In fact, a systematic review by Jancelewicz et al. [13] noted a decrease in recurrence rates from 50% prior 2008 to 25% after 2008, suggesting that surgical skills were improving. On the other hand, when compared with open surgery, minimally invasive CDH repair has been associated with lower mortality rate. Nevertheless, these results have been related with selection bias, i.e., groups where minimally invasive repair may included less severe cases [11]. In 2017, Putnam et al. [14] stratified CDH patients by risk and demonstrated that although minimal invasive repair was associated with shorter hospital stays and fewer adhesive small bowel obstruction, it was still associated with higher recurrence rates. Thus, the benefits of minimal invasive surgery in CDH patients are still inconclusive. Thoracoscopic repair of CDH is not a routine treatment for every neonate and it is important to select suitable cases for this approach [11, 14].

A variety of prosthetic materials has been developed for PR [15]. Polytetrafluoroethylene (PTFE; Gore-Tex^®^), a synthetic non-absorbable polymer, is a durable and resistant material and therefore considered the gold-standard for diaphragmatic replacement [16]. However, it is associated with poor tissue integration and high recurrence rates associated with the subject growth [17–19]. Other synthetic materials, such as Seamdura^®^ are also associated with high recurrence rates [20]. Several biological materials were applied in PR, such as small intestine submucosa (SIS, Surgisis^®^) [17–19, 21], autologous biosheets [20], decellularized diaphragms [22, 23], acellular collagen matrices [18, 24–27], acellular dermal matrix (AlloDerm) [28] and porcine dermal collagen (Permacol^®^) [29]. Despite advantages such as low immunogenicity and tissue remodeling, absorbable biomaterials are still a limited choice for diaphragmatic replacement due to patch thinning and incomplete tissue ingrowth and consequently a higher-than-desired re-herniation rate. Blend materials with synthetic and biological components were also proposed for CDH [30–33]. Although studies show better tissue integration, recurrence rates are still sub-optimal.

Aligned on the therapeutic potential of extracellular matrix (ECM)-based membranes [34, 35], two different patches were analyzed in this study: a synthetic patch that mimics ECM morphology, and a biological membrane that preserves both ECM architecture and components. The synthetic patch is a fibrous membrane made of polycaprolactone (PCL), randomly organized in a mesh-like structure, fabricated using the electrospinning technique (PCLem) [36]. Based on previous studies with diverse cell types [37–39], it is hypothesized that PCLem should encourage host angiogenesis, cell proliferation, migration and differentiation, eventually replacing the graft with constructive connective tissue. The biological membrane is a decellularized human chorion membrane (dHCM), resulting from the decellularization of human chorion membrane, as previously described [40]. It is a compact and acellular membrane that was shown to be biocompatible both in vitro and in vivo, and to be integrated by host tissue [41].

**Fig. 1:**
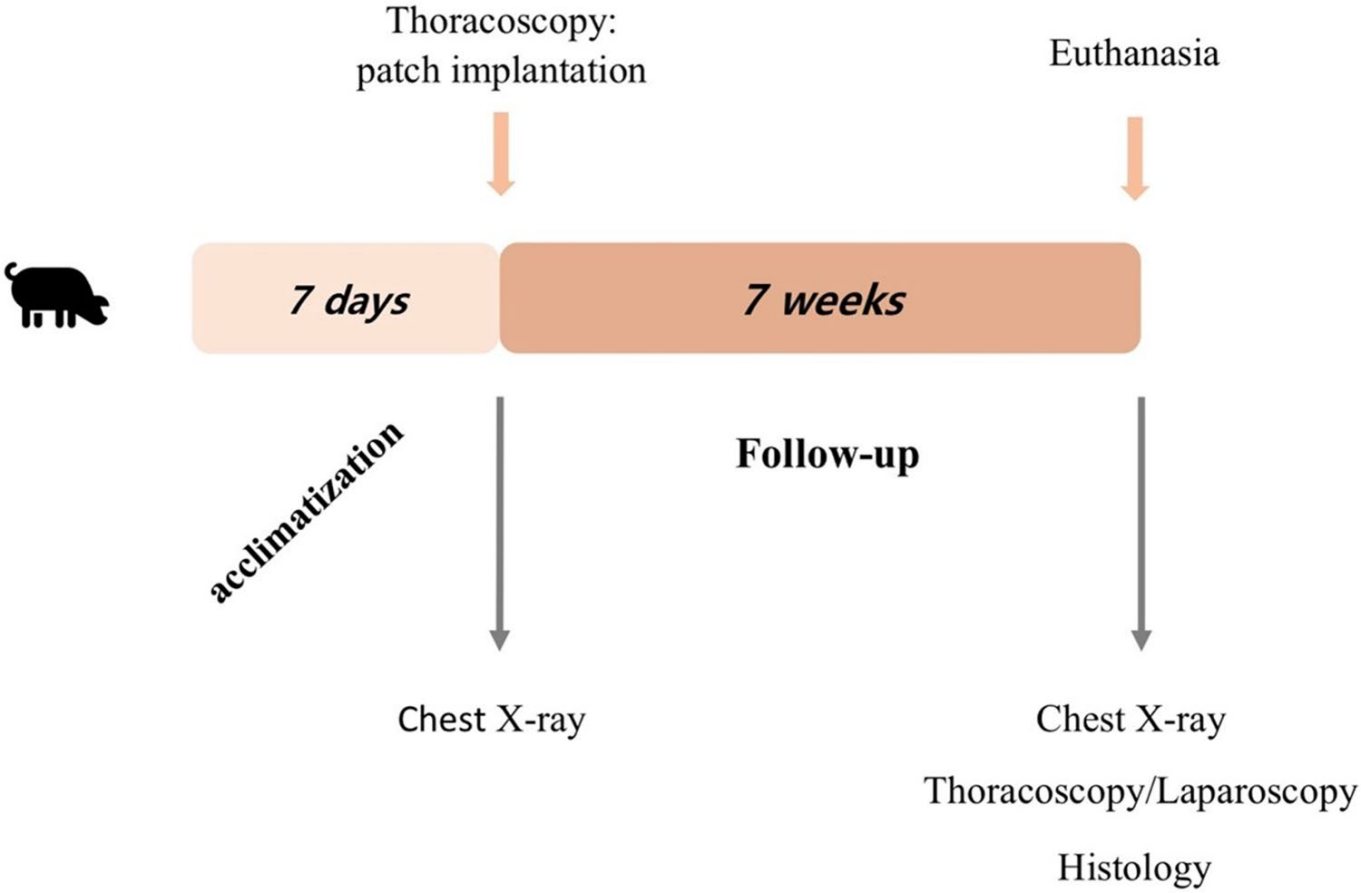
Experimental timeline: Farm male pigs were acclimatized for 7 days and then submitted to a chest X-ray and thoracoscopic surgery for patch implantation. Seven weeks post-surgery, animals were submitted to a second chest X-ray, thoracoscopy, laparoscopy and were euthanized

Therefore, the aim of this study is to mimic the condition of neonatal PR in CDH by assessing a novel diaphragmatic hernia model using thoracoscopic techniques in sexually immature young farm pigs. Moreover, two novel membranes (PCLem and dHCM) will be compared to the clinical gold-standard (Gore-Tex^®^) for diaphragmatic hernia treatment (Fig. 1).

### Methods

This study was performed following the EU Directive 2010/63/EU and approved by the Animal Ethics Committee of the institution where the study was performed (ORBEA EM.ICVS-I3Bs_010/2019).

### Patch preparation

Gore-Tex^®^ Soft Tissue Patch was purchased from W. L. Gore & Associates (Delaware, USA). PCLem was prepared using a solution of 20% PCL with a solvent mixture of chloroform and dimethylformamide (8:2 ratio). Then, the PCL solution was electrospun by applying a voltage of 15.5 kV, a needle tip to ground collector distance of 20 cm, and a flow rate of 2 mL/h. After the complete processing of 1 mL of PCL solution (30 min), PCLem was allowed to air dry for 1 day. dHCM were obtained from human placentas as previously described [40].

### Animal care and use

Fourteen farm male pigs (*Sus scrofa domesticus*) with approximately 6 weeks of age (6.54 ± 0.57 kg body weight) were acclimatized for 7 days. Animals were housed in groups of 2 or 3 animals per pen (24 ± 2 °C and automatic air renewal). Dry pellet food was provided once a day and water was available ad libitum. Animals were randomly assigned to 3 different experimental groups of 4 animals each, determined by the type of patch used during thoracoscopic diaphragmatic repair (Gore-Tex^®^, PCLem and dHCM). Two additional animals were used as age and weight-matched controls and submitted to thoracoscopy without creation of diaphragmatic defect (Sham group).

### Chest X-rays

Pre-operatively and at the end of the protocol (euthanasia), all animals were submitted to a chest X-ray evaluation under sedation to document lung pathology (i.e., atelectasis, infection), hernia recurrence or diaphragmatic eventration, and skeletal deformations such as scoliosis or thoracic wall deformities.

### Anesthesia and analgesia

All surgical procedures were performed under general anesthesia with endotracheal intubation and mechanical ventilation. Eight hours fasting period was performed. Animals were anesthetized with a combination of ketamine (20 mg/kg, intramuscularly (IM)), xylazine (2 mg/kg), and atropine (0.04 mg/kg, IM), followed by propofol (4 mg/kg, intravenously (IV)) and maintained with continuous propofol infusion (20 mg/kg.h, IV) and buprenorphine (0.05 mg/kg). Additional intra and post-operative analgesia was provided by non-steroidal anti-inflammatory Meloxicam (0.4 mg/kg, IM). Maintenance of water and electrolyte balance was provided by intravenous administration of isotonic fluids (10 ml kg^−1^ h^−1^, IV). An electronic microchip transponder was implanted at each animal for individual identification (*LifeChip, BioThermo*) through a portable reader (*SURESense, SUREFlap*).

### Surgical technique

**Fig. 2:**
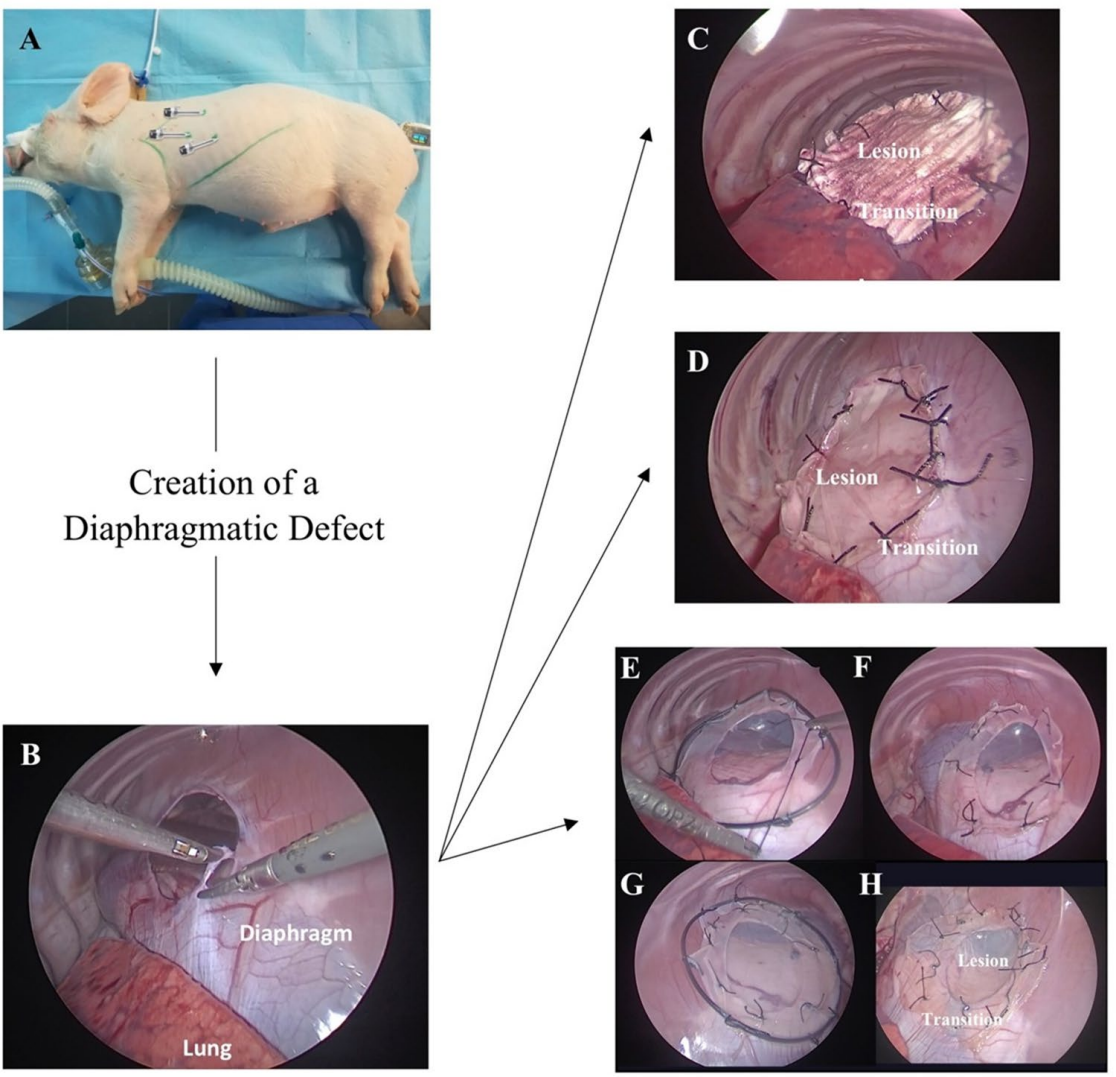
Thoracoscopic defect and repair of the diaphragm. Representation of trocars site (**A**). Thoracoscopic creation of a diaphragm defect (**B**). Repair of the diaphragm defect with Gore-Tex^®^ (**C**), PCLem (**D**) and dHCM (**E–H**)

The order of surgeries for different patch-types was randomized as well as the animals subjected to the different treatment groups. All surgical interventions were performed by the same surgeon under sterile conditions and continuous cardiorespiratory monitoring. Animals were positioned in right lateral decubitus, with the left foreleg retracted cranially. A nasogastric tube was placed. A 5 mm port was placed in the 6^th^ intercostal space aligning with the shoulder joint, for introduction of a 5 mm 30° telescope. Two working ports were placed in the 7^th^ intercostal space under endoscopic vision, one 3 mm port along a line passing through the tip of the left scapula and the other 5 mm port approximately 5 cm from the left costal arch, allowing access to the diaphragm (Fig. 2A). Capnothorax was established with an inflation rate of 1 L/min and maintained at a maximum pressure of 3 mmHg. A left posterolateral type A diaphragmatic defect of 5 cm diameter was performed using a 3 mm scissor (Fig. 2B). Each patch was placed intra-thoracic via a 5 mm port and fixed using non-absorbable silk 4/0 or 5/0 simple interrupted sutures. While placing the dHCM and Gore-Tex^®^ patches, care was taken to place the previously marked side facing the abdominal cavity, since it has a lower pore size intended to minimize tissue attachment. Patches were tailored to the size and shape of the diaphragmatic defect. Two dHCM membranes were used in each pig. The dHCM patch was sutured to a plastic o-ring, allowing the membrane to unfold when inside the thorax and adjust to the defect area (Fig. 2E). After suture of dHCM to diaphragmatic rims, the o-ring was removed (Fig. 2F). Then a second dHCM membrane, also sutured to a plastic o-ring was placed above the first one (Fig. 2G), its sutures were intercalated, and the o-ring was removed (Fig. 2H). Trocar-placement wounds were closed in layers using 2/0 polyglactin suture (Vicryl^®^; Ethicon, New Jersey, USA), and a running subcuticular 4/0 poliglecaprone suture (Monocryl^®^; Ethicon). Wounds were covered with a t-shirt to prevent scratching and biting during the post-operative period.

### Post-operative care

All animals were monitored in the operative room until the occurrence of righting reflex. Antibiotic coverage was provided by an 8-day course of enrofloxacin (5 mg/kg; IM). Post-operatively, animals were closely checked for any signs of complication, distress, behavior changes, anorexia, or weight loss. When any of these signs was present, human endpoints were applied to avoid suffering.

### Euthanasia and necropsy evaluation

Pigs were euthanized after a postoperative period of 7 weeks. Before euthanasia, all animals underwent a thoracoscopic and laparoscopic exploration to evaluate adhesion formation and the macroscopic condition of the patches. Euthanasia was performed by administration of an overdose of pentobarbital (2000 mg/kg, IV) after administration of ketamine (20 mg/kg, IM) and xylazine (2 mg/kg, IM). In each animal, both hemidiaphragms were collected for further histologic evaluation. The right hemidiaphragm was used as control. In the patch repair groups, 6 tissue samples were collected from the left diaphragm: 3 in the former patch area (lesion zone) and 3 in the patch-diaphragm interface area (transition zone). Additionally, 6 tissue samples were collected from the right hemidiaphragm: 3 from the muscular area and 3 from the tendon area of the diaphragm. Same procedure was applied the Sham group.

### Outcome measurements

Pre- and post-operative data were collected, such as date of surgery/euthanasia, weight at surgery/euthanasia, intraoperative time, post-operative complications and/or recurrent herniation, rib synostosis, scoliosis or wall deformation, and intrathoracic and intrabdominal adhesion formation. A Scoring system for newly formed diaphragmatic tissue was adapted from Gonzalez et al. [17]. Adhesion formation was assessed by thoracoscopy and laparoscopy at euthanasia and during necropsy. Patches were easily identifiable, therefore macroscopic adhesion was non-blinded graded. Inflammation, angiogenesis, and fibrosis were scored through Hematoxylin–eosin (HE) and Masson’s trichrome (MT) slides (Table 1).

**Table 1:** Scoring system

Score	Adhesion formation	Inflammation	Angiogenesis	Fibrosis
0	None	None	None	None
1	Minimal adhesions that could be freed by gentle blunt dissection	Minimal	Minimal	Minimal
2	Moderate adhesions that could be freed by aggressive blunt dissection	Mild	Mild	Mild
3	Dense adhesions that required sharp dissection	Moderate	Moderate	Moderate

Adapted scale from Gonzalez et al. [16] used for assessing and grading macroscopic adhesion by thoracoscopy and laparoscopy, and inflammation, angiogenesis, and fibrosis on hystology

### Histology

Samples were fixed in 10% formalin, embedded in paraffin and cut in 5 μm sections. HE and MT staining were performed. At least five fields per slide were randomly analyzed and scored (Table 1) by one operator (AL-F) who was blinded to the study groups and outcome at the time of the evaluation. An Olympus System Microscope was used to photograph the stained section.

### Immunohistochemistry

Paraffin-embedded sections were incubated with rabbit anti-alpha smooth muscle actin (anti α-SMA; 1:50, #ab5695; Abcam, Cambridge, UK), rabbit anti-CD31 (1:50, #ab28364; Abcam) and mouse anti-CD105 (1:100, #MCA1557F; BioRad, California, USA) for 2 h at room temperature. A secondary antibody from the R.T.U. VECTASTAIN^®^ Universal ABC Elite^®^ Kit (#PK-7200, Vector Laboratories, UK) was used in accordance with manufacturer’s instructions. Incubation was revealed using Peroxidase Substrate Kit (DAB) (#SK-4100, Vector Laboratories). Sections were counterstained with hematoxylin and mounted in an aqueous mounting medium. Slides were observed in an optical microscope with a coupled camera (DM750, Leica, Wetzlar, Germany).

### Statistical analysis

Results analysis was performed using GraphPad Prism 7 (GraphPad Software, Inc. 2016). Comparisons between groups were performed by One-way ANOVA followed by Dunn’s multiple comparison test. To compare the survival distributions of the groups, Logrank test was used. A *p* < 0.05 was considered statistically significant.

## Results

### dHCM is associated with a higher operative time

Thoracoscopic defect creation and diaphragmatic repair was performed without any technical difficulty in all groups (Fig. 2). One animal (dHCM group) died during surgery due to anesthetic complications and was excluded from the study (*n* = 13). Overall operative time was 178.9 min. However, intraoperative time (Table 2) was higher in the dHCM (240.0 min) than in the PCLem (172.5, *p* = 0.203) and Gore-Tex^®^ (156.3 min, *p* = 0.025) groups. During the surgeries, 3 pigs (G3, P2, P3) presented intrathoracic signs of previous pneumonia, such as adhesions and mucus. Nevertheless, those issues did not influence surgery duration (Table 2). One pig (P3) presented respiratory distress at the end of the surgery due to aspiration, as well as difficulty in the left foreleg locomotion in the post-operative period (probably due to brachial plexus lesion during surgery). No other types of intra- or post-operative complications occurred.

**Table 2:** Descriptive table summarizing different outcome measures, such as surgery duration (minutes), follow-up time (days), recurrence rate (%), weight gain from surgery to euthanasia (kg), and adhesion formation grade at time of death per animal

Experimental groups	Animals	Operative time (min)	Mean operative time (min)	~ 7 weeks follow up	Follow-up time (days)	Recurrence	Recurrence rate (%)	Weight gain (Kg)	Adhesions grade (score 0–3)	Mean Adhesions grade (score 0–3)
Sham group (*n* = 2)	S1	150	145.0	Yes	52	No	0	21.9	2	1.5
	S2	140		Yes	51	No		24.7	1	
Gore-Tex^®^ group (*n* = 4)	G1	130	156.3	Yes	50	No	0	34.6	3	2.3
	G2	180		Yes	47	No		21.2	2	
	G3	150		Yes	48	No		31.2	2	
	G4	165		Yes	63	No		36.1	2	
PCLem group (*n* = 4)	P1	180	172.5	No	45	Yes	50	15.9	2	2.3
	P2	150		Yes	47	No		29.4	2	
	P3	180		Yes	48	No		19.9	3	
	P4	180		No	33	Yes		11.4	2	
dHCM group (*n* = 3)	D1	300	240.0	No	14	Yes	100	6.1	1	1.3
	D2	210		No	27	Yes		10.0	1	
	D3	210		No	23	Yes		9.4	2	

### PCLem and dHCM are associated with hernia recurrence

From the total 13 animals included in this study, 8 animals (2 from Sham, 4 from Gore-Tex^®^ and 2 from PCLem groups) survived until the end of the experimental timepoint of (7 weeks). Five animals (3 from dHCM and 2 from PCLem groups) developed sudden dyspnea and anorexia 14, 23, 27, 33, and 45 days after surgery, respectively, and humane endpoints were applied. In these subjects, complete or partial detachment of the patch and associated herniation of the stomach into the left hemithorax was found during necropsy. Regarding the remaining animals, chest X-ray performed at the end of the follow-up period revealed a normal diaphragm, with no evidence of eventration, hemithorax volume reduction, lung pathology, scoliosis or chest wall deformities when compared to pre-operative imaging (data not shown). The hernia recurrence rate was 0% in Gore-Tex^®^ group, 50% in PCLem group and 100% in dHCM group (Table 2). These results were reflected in follow-up time and weight gain of the animals (Table 2). For the animals that make it to 7 weeks (S1, S2, G1, G2, G3, G4, P2, P3), the overall weight gain was 27.4 kg, corresponding to a triplication of the original weight (mean: 8.9 kg, data not shown). As previously stated, at the time of euthanasia all animals underwent thoracoscopy and laparoscopy. Although thoracic scars were unremarkable, 5 animals (3 from Gore-Tex^®^—G2,G3,G4—and 2 from PCLem—P2, P4—groups) presented post-operative calcification adjacent to the 6th, 7th and 8th ribs, where trocars were placed (data not shown). Regarding adhesion formation, though no statistically significant differences were observed between the 3 treatment groups (*p* = 0.121), PCLem and Gore-Tex^®^ groups had a similar mean score of 2.3, while dHCM group was associated with a lower level of adhesion formation (mean score of 1.3), similar to the one observed on Sham group (mean score 1.5) (Table 2). Gore-Tex^®^ was associated with adhesions to the lung and the omentum, despite its dual surface structure and correct placement. PCLem group presented gastric adhesions and spread and tenacious adhesions both in thorax and abdomen. dHCM adhesions were minimal and limited to trocar level. In Sham group, adhesions were only observed at thoracic incision points and between lung and diaphragm.

### PCLem and dHCM are associated with better tissue integration

**Fig. 3:**
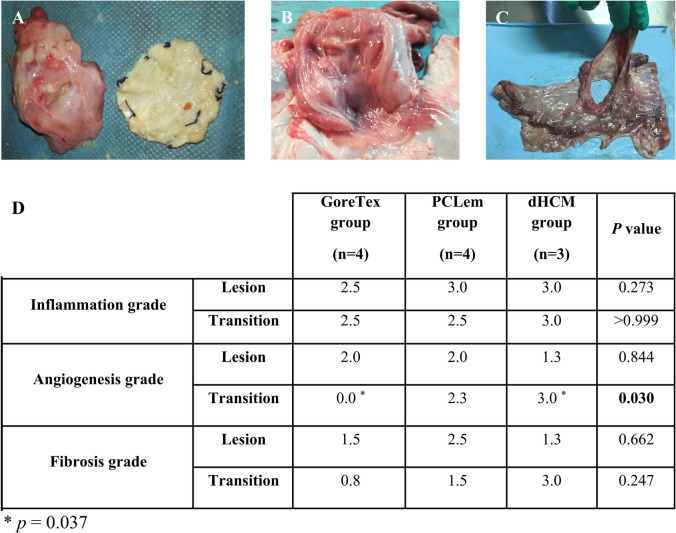
Macroscopic and microscopic evaluation of new diaphragmatic tissue. Top view of Gore-Tex^®^ (**A**), PCLem (**B**) and dHCM (**C**) after follow-up time. Comparison of inflammation, angiogenesis and fibrosis mean scores (score 0–3) between groups, defined by the histologic analysis of hematoxylin/eosin and Masson’s trichrome staining. Two different tissue areas were analyzed, lesion site that corresponds to the patches and transition that represents the native diaphragm at the site of the sutures (**D**)

At euthanasia, it was possible to observe that the sutures detach from the diaphragm rim in PCLem and dHCM groups but not in Gore-Tex^®^ group. Gore-Tex^®^ patches appeared virtually unchanged and covered with a fibrotic capsule (Fig. 3A). The thoracic surface of the implanted patch was partially agglutinated to the lung in 3 animals (G1, G2 and G4), and the abdominal surface was agglutinated to the omentum in all animals (*n* = 4). Moreover, one animal (G3) presented serous fluid accumulation between the layers of the regenerated fibrotic tissue. Another animal (G1) had intrathoracic signs of infection/inflammation and a capsulated abscess in the area of Gore-Tex^®^ implantation. Nevertheless, this animal never presented clinical signs of infection during the follow-up period, presenting a weight gain of 34.6 kg, higher than mean weight in the Gore-Tex^®^ group (30.8 kg), and reflecting an initial body weight quintuplication in 50 days. In contrast, in the PCLem and dHCM groups, the patches could not be identified. In the PCLem group, it was replaced by a floppy connective tissue (Fig. 3B). In both animals submitted to humane endpoints (P1 and P4), adhesions between lung, patch and stomach were observed. Regarding the other two animals in the PCLem group (P2 and P3), one presented partial agglutination between the thoracic surface of the implanted patch and the lung (P2), while the second had dense adhesions between abdominal organs (peritonitis), a bulging stomach and compact adhesions between lung, patch, and stomach (P3). Interestingly, no weight loss was observed in this last animal, presenting a weight gain of 19.9 kg which corresponds to a 200% increase from the animal initial weight (10.1 kg). In the dHCM group, the patch was replaced by a vascularized and floppy regenerated membranous tissue (Fig. 3C). One pig (D3) presented the thoracic surface of the patch partially agglutinated with the lung.

After macroscopic analysis, hematoxylin/eosin and Masson’s trichrome staining were performed to analyze inflammation, angiogenesis, and fibrosis grade. As previously described, a scoring system was used (Table 1). A summary of the results is presented in Fig. 3D. Although no statistically significant differences were observed (*p* = 0.273), inflammation was slightly lower in the lesion site in the Gore-Tex^®^ group (Fig. 3D). This slight decrease in inflammation grade was also observed in the transition zone in Gore-Tex^®^ and PCLem groups in comparison to dHCM group, which had the higher score for inflammation in both sites (lesion and transition). Inflammation scores did not change from lesion to transition site in Gore-Tex^®^ and dHCM groups. In PCLem group, a decrease is observed between the two sites. Lymphoplasmacytic inflammation was observed in all groups with presence of some neutrophils, macrophages, and foreign-body giant cells. Regarding angiogenesis (Fig. 3D), no statistically significant differences were observed in the lesion site, although dHCM group presented a lower score. In the transition zone, dHCM group had the higher score for angiogenesis and a statistically significant difference was observed when compared with Gore-Tex^®^, where angiogenesis was not observed (*p* = 0.037). While PCLem and dHCM groups shown an increase in the score from lesion to transition site, in the Gore-Tex^®^ group a decrease is observed. This decreasing trend from lesion to transition site was maintained for fibrosis grade in Gore-Tex^®^ and PCLem groups, in contrast to the dHCM group that shows an increase (Fig. 3D). At the lesion site, fibrosis grade is higher in PCLem group and lower in dHCM group. However, in the transition zone, dHCM group shows the highest score and Gore-Tex^®^ group the lowest. Nevertheless, no statistically significant differences were observed for fibrosis in both tissue zones (lesion and transition).

**Fig. 4:**
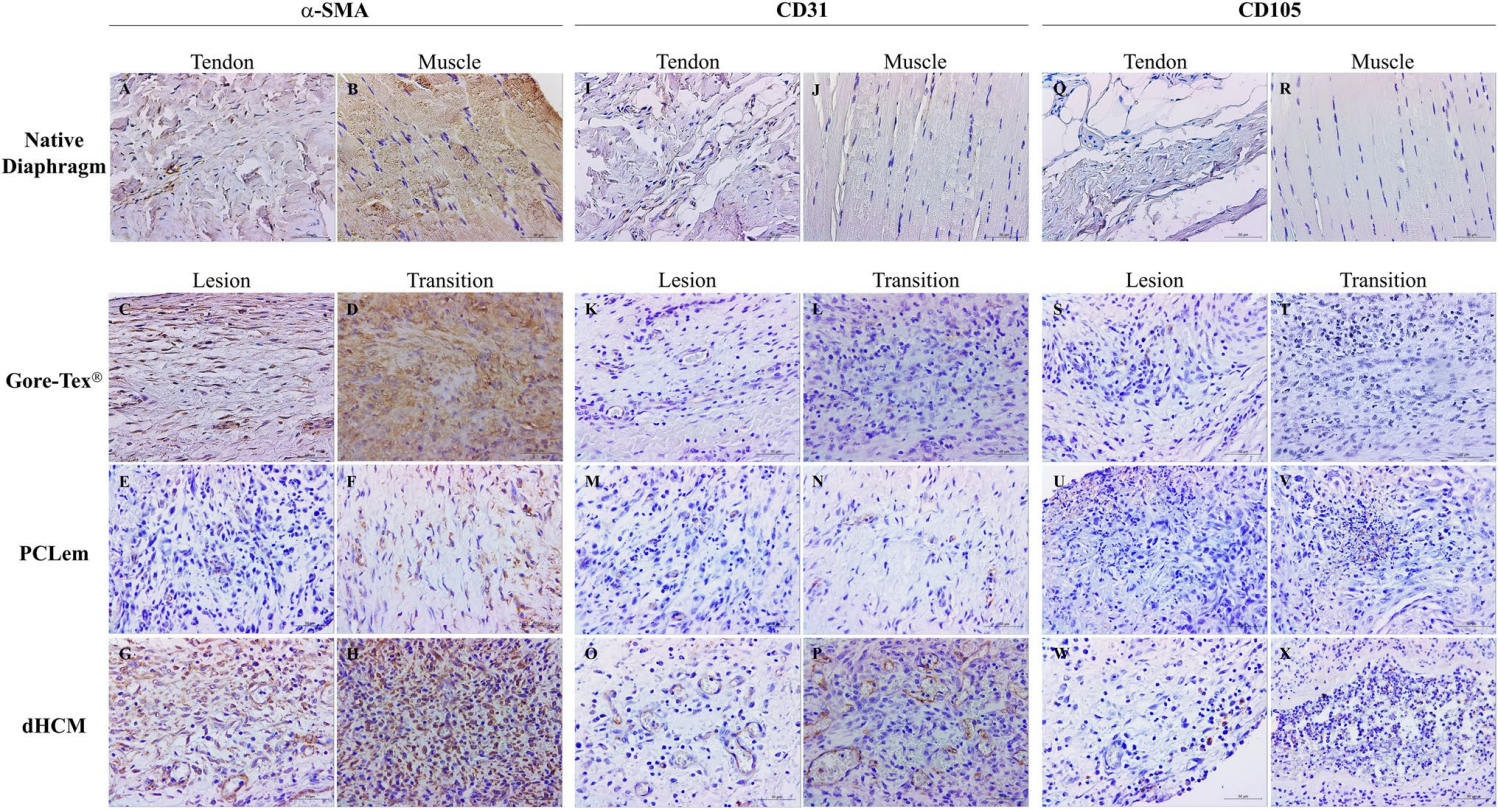
Immunohistochemistry for α-SMA, CD31 and CD105. Representative sections of α-SMA, CD31 and CD105 immunostaining of the native diaphragm, in both tendon (**A**, **I** and **O**, respectively) and muscle (**B**, **J** and **R**, respectively). Patch samples were divided into two distinct zones: lesion and transition (Fig. 2C, D and H). Representative sections of α-SMA, CD31 and CD105 for Gore-Tex^®^ (**C**–**D**, **K**–**L**, **S**–**T**, respectively), PCLem (**E–F**, **M–N**, **U**–**V**, respectively) and dHCM (**G**–**H**, **O**–**P**, **W**–**X**, respectively) groups

To further assess the tissue regeneration, immunohistochemistry was performed for muscle (α-SMA), endothelium (CD31) and stem cells (endoglin; CD105) (Fig. 4). A positive immunostaining for α-SMA was observed in all groups (Fig. 4C–H). However, this immunostaining was more intense in the Gore-Tex^®^ group (Fig. 4C, D) and less intense in the PCLem group (Fig. 4E, F). In all conditions, the transition zone (Fig. 4D, F and H) presented a higher immunostaining for α-SMA when compared to the lesion zone (center). Moreover, α-SMA-positive blood vessels were observed in dHCM in both zones. Regarding CD31 immunostaining, although it was present in the three groups in both zones (lesion and transition) (Fig. 4K–P), it was more intense in dHCM transition zone (Fig. 4P). CD105 positive immunostaining was only observed in inflammatory infiltrates, regardless patches, and zones (lesion or transition) (Fig. 4S–X).

## Discussion

In this study, a novel diaphragmatic hernia animal model was implemented for the first time, using thoracoscopic techniques in sexually immature farm pigs, to study the performance of three different membranes in the treatment of large diaphragmatic defects.

To mimic a diaphragmatic hernia, circular diaphragmatic defects with 5 cm diameter were created. Since this diaphragmatic defect would be fatal, as confirmed in the pilot study (data not shown), Sham animals were only submitted to thoracoscopy without creation of the defect. Although the rapid growth rate of the animals being comparable to human childhood, the animal model missed the hypoplastic lung parenchyma characteristic of CDH. Furthermore, to allow patch placement (A defect), diaphragm rims were maintained, excluding the clinical cases of CDH where the patch is needed (large B, C and D defects) [1]. Despite the described limitations, this model gives insights on how new membranes would behave in a setting of thoracoscopic diaphragmatic hernia repair, opening new research lines for CDH.

As it would be expected for animals that have been bred predominantly for meat production, pigs’ musculature was massive. Prior to this study, a pilot study with 7 animals (1 Sham, 2 Gore-Tex^®^, 2 PCLem and 2 dHCM) was performed to allow the surgeon become familiar with the handling properties of each material and to perfect both operative technique and post-operative management. This allowed surgeries be performed without any technical difficulty in all groups (Gore-Tex^®^, PCLem and dHCM). However, dHCM group was associated with a higher operative time, due to the handling of dHCM fixed in o-rings and to the use of two membranes per animal. Without o-rings it was impossible to stretch dHCM in the thoracoscopic space; moreover, to guarantee the closure of the defect, two dHCM were necessary per pig. During this study, the overall pig’s weight gain was 20.5 ± 10.9 kg in 42.2 ± 13.8 days, being in accordance with farm pigs growth curve [42]. Probably due to the small rim of muscle left during the diaphragm excision, longitudinal follow-up assessments (chest X-rays performed before patch implantation and at the end of the study) demonstrated that no animal presented musculoskeletal sequelae, which could have impacted the ingrowth of the patch and reduce the tension on the chest wall, influencing the results. The grade of adhesions—the result of the inflammatory response to injury in the peritoneal space [43]—varied in accordance with the type of patch implanted, being higher in Gore-Tex^®^ and PCLem groups than in dHCM and Sham groups.

Diaphragm manipulation with prothesis implantation is always necessarily followed by an inflammatory process that ultimately leads to inclusion, degradation, or rejection of the prothesis. As foreign bodies, all patches will trigger a process of inflammation, fibrosis and neovascularization [44, 45]. As expected [19], a lymphoplasmacytic inflammation with the presence of some neutrophils, macrophages, and foreign-body giant cells was observed in all conditions. In general, fibrosis grade was comparable between the three groups. However, a slight but not statistically significant difference was observed between Gore-Tex^®^ and dHCM groups in the transition zone. Similar results had been reported in studies using Gore-Tex^®^ and Surgisis^®^ [17, 18]. As previously reported [17–19], Gore-Tex^®^ presented poor tissue integration and its surface was covered by a fibrotic capsule which was likely due to a foreign body response. In contrast, dHCM was replaced by a vascularized connective tissue, preventing the identification of the membrane, as already described for natural membranes [17, 18]. And the surface of PCLem was replaced by a floppy connective tissue making the membrane no longer identifiable, neither macro nor microscopically. This was not in accordance with Zhao et al. [30] that used aligned PCL/collagen type I hybrid scaffolds for diaphragmatic muscle reconstruction. In that study, the scaffold was identifiable until the 4th month (approximately 112 days). Nevertheless, Zhao et al. [30] used a different animal model (female Lewis rats with 14 weeks old) which may influence the degradation rate of the material [30]. Regarding vascularization, α-SMA positive blood vessels were observed in dHCM in both lesion and transition zones, suggesting a functional tissue vascularization at 21.3 ± 6.7 days (3 weeks) post-surgery which was also corroborated by CD31 immunostaining (endothelial marker) and angiogenesis scores. For the PCLem, the low CD31 immunostaining together with the high CD105 stanning intensity suggest a neovascularization preparation [46–48] after 43.3 ± 7.0 days (6 weeks). This was already observed in a similar study using aligned PCL/collagen type I hybrid scaffold [30], where visible graft vascularization started to be observed on the 2nd month and increased until the 6th month. Concerning muscle formation, no muscle layers were observed in the implanted areas after 40.5 ± 14.4 days. This result was already expected since muscle formation is only observed in implanted areas after 24 weeks (168 days) [33]. In further studies, longer follow-up times are needed to assess muscle formation.

In general, PCLem and dHCM groups were associated with a better tissue integration than Gore-Tex^®^, but also with a lesser follow-up time related with hernia recurrence (50% in PCLem and 100% in dHCM). It was hypothesized that, while the patch was degraded, the replacing tissue was weaker than the native one and not able to prevent recurrence when it was subjected to tension related to the rapid growth of the animal. Thus, the pig’s rapid growing curve, together with a fast patch degradation, could have influenced the recurrence rate. This was not expected since it was demonstrated that, when placed subcutaneously in mice, dHCM only started to be integrated by host tissue at day 28 [41]. So, it is important to understand if the rapid patch degradation is related with pig’s inflammatory response and collagen metabolism, or if it will persist in other animal models, or if it is related with the implantation site. In future studies, more dHCM layers should be used in accordance with what is done with Surgisis^®^ (4–8 ply) [17–19]. Despite no recurrent herniation was observed in the Gore-Tex^®^ group, in 2 cases the patch was partially detached from the diaphragmatic rim.

While minimal invasive surgery, such as thoracoscopic, has been associated with higher recurrence rates when compared with open surgery [14], the benefits of one technique over the other remain inconclusive [11, 14]. Regarding Gore-Tex^®^ patches, a recurrence rate of 0% was observed in our study. Nevertheless, in a study using open abdomen surgery for diaphragmatic hernia treatment in Yucatan pigs, the recurrence rate of PTFE-based membranes was 30% [17]. In the same study, a natural membrane was used (SIS) that presented a recurrence rate of 0% [17], in contrast to our results (100% recurrence rate dHCM). Although this may be associated with the surgical technique, it can also be related with the number of membrane layers used, as previously stated. While two dHCM layers were used in this study, in Gonzalez et al. [17], eight layers of SIS were used. Another study using open abdomen surgery for the treatment of diaphragmatic hernia using cryopreserved and decellularized human hemidiaphragm in a dog model, demonstrated a herniation recurrent rate of 0% after 6 months of follow-up [49]. Nevertheless, the nature and composition of the patches is different from the ones used in this study, which may have a greater influence in the study outcomes than the surgical procedure alone.

Lastly, one major concern about this study is the difference between forces acting on the pig diaphragm when compared to human, since animals spend the majority of their time walking on “all fours”, thereby possibly influencing different stress patterns on the grafts and altered frequency of herniation.

## Conclusions

In this work, sexually immature farm pigs and thoracoscopic techniques were used to establish a new diaphragmatic hernia model. This growing large animal model was used to compare two new membranes, one synthetic (PCLem) and another biological (dHCM) against the clinical gold standard (Gore-Tex^®^) for diaphragmatic hernia repair. In summary, when compared to Gore-Tex^®^, PCLem and dHCM were associated with better tissue integration and neovascularization, suggesting that the patches would grow with the subject. Nevertheless, PCLem and dHCM were also related with a higher hernia recurrence (50% and 100%, respectively), when compared to Gore-Tex^®^ (0%). Additional long-term studies using PCLem and dHCM would be determinant in the  creation of a solution that allows tissue regeneration in situ without hernia recurrence. Moreover, improvements in animals’ models and membrane design are needed.

## Supplementary Information

Below is the link to the electronic supplementary material.

Supplementary file1 (DOCX 357 KB)

## Data Availability

Data supporting this study are included within the article and/or supporting materials, and it is available upon reasonable request.

## Abbreviations

CDH: Congenital diaphragmatic hernia
ECM: Extracellular matrix
PCL: Polycaprolactone
dHCM: Decellularized human chorion membrane
CDHSG: Congenital diaphragmatic hernia study group
PR: Patch repair
