# Supplementary material for: Diaphragmatic hernia repair porcine model to compare the performance of biodegradable membranes against Gore-Tex®
Source: Pediatr Surg Int. 2023 Nov 24;40(1):7. doi: 10.1007/s00383-023-05584-x (PMC10673990; doi:10.1007/s00383-023-05584-x)
Supplement: Supplementary file 1 — Supplementary file1 (DOCX 357 KB) [file 383_2023_5584_MOESM1_ESM.docx]

**Diaphragmatic hernia repair porcine model to compare the performance of biodegradable membranes against Gore-Tex^®^**

Marianna Scuglia a, b, ^&^ , Laura. P. Frazão b, c, ^&^, Alice Miranda a, b, Albino Martins b, c, Joana Barbosa-Sequeira a, b, e, Diana Coimbra a, b, Adhemar Longatto-Filho a, b, f, g, Rui L. Reis b, c, Cristina Nogueira-Silva a, b, h, Nuno M. Neves b, c *, Jorge Correia-Pinto a, b, d

a Life and Health Sciences Research Institute, School of Medicine, University of Minho, Braga, Portugal; b ICVS/3B’s – PT Government Associate Laboratory, Braga/Guimarães, Portugal; c I3B’s – Research Institute on Biomaterials, Biodegradables and Biomimetics of University of Minho: 3Bs Research Group, Guimarães, Portugal; d Department of Pediatric Surgery, Hospital de Braga, Braga, Portugal; e Department of Pediatric Surgery, Centro Materno Infantil do Norte, Centro Hospitalar Universitário do Porto, Porto, Portugal; f Department of Pathology (LIM-14), University of São Paulo School of Medicine, São Paulo, Brazil; g Molecular Oncology Research Center, Barretos Cancer Hospital, Barretos, São Paulo, Brazil; h Department of Obstetrics and Gynecology, Hospital de Braga, Braga, Portugal.

^&^ Authors Marianna Scuglia and Laura P. Frazão have contributed equally to the work.

**Corresponding Author:**

*Nuno M Neves, e-mail: nuno@i3bs.uminho.pt; Address: 3B’s Research Group, AvePark, Parque de Ciência e Tecnologia, Zona Industrial da Gandra, 4805-017 Barco, Guimarães, Portugal. Tel: +351-253-510905 (Direct) or +351-253-510900; Fax: +351-253-510909

**
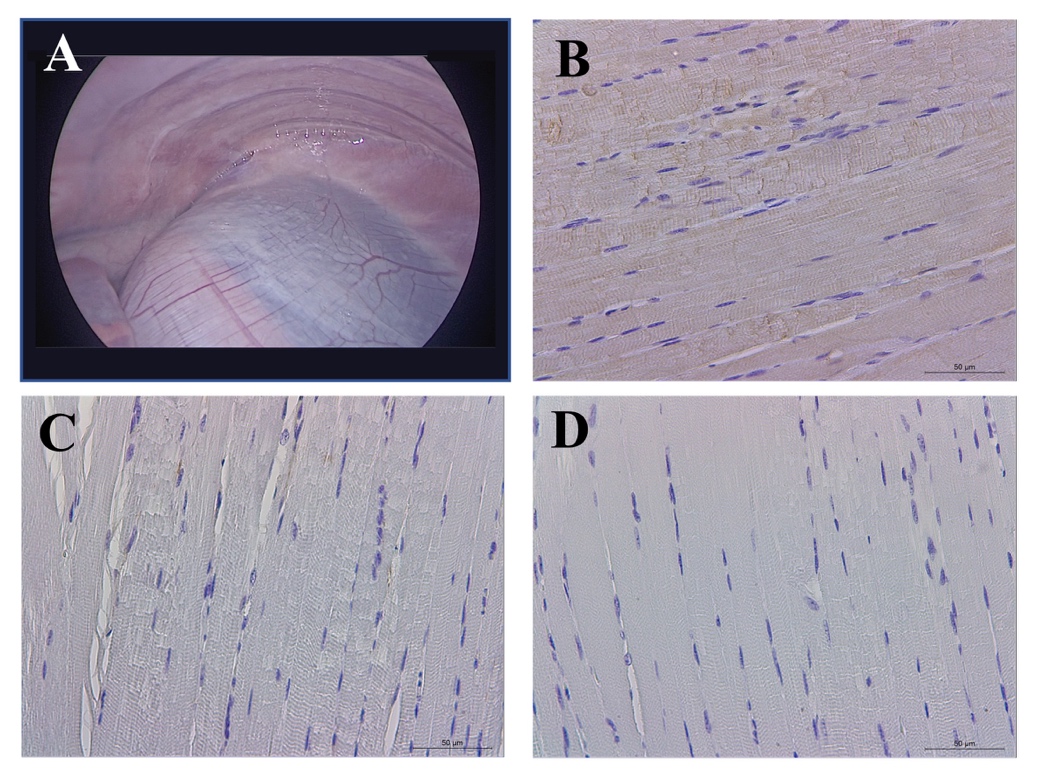
**

**Figure S1 - Sham animals’ characterization.** Thoracoscopic sham surgery, without defect creation **(A)**.

Representative sections of α-SMA **(B)**, CD31 **(C)** and CD105 **(D)** staining of diaphragm from sham animals
